# *γδ* T/Interleukin-17A Contributes to the Effect of Maresin Conjugates in Tissue Regeneration 1 on Lipopolysaccharide-Induced Cardiac Injury

**DOI:** 10.3389/fimmu.2021.674542

**Published:** 2021-04-26

**Authors:** Yi Yang, Xin-Yu Li, Lin-Chao Li, Ji Xiao, Yin-Meng Zhu, Yang Tian, Yong-Mao Sheng, Yan Chen, Jian-Guang Wang, Sheng-Wei Jin

**Affiliations:** ^1^ Department of Anesthesia and Critical Care, The Second Affiliated Hospital and Yuying Children’s Hospital of Wenzhou Medical University, Wenzhou, China; ^2^ Department of Biochemistry, School of Basic Medical Sciences, Wenzhou Medical University, Wenzhou, China

**Keywords:** neutrophil, interleukin-17A, lipopolysaccharide, cardiac injury, γδ T cells

## Abstract

The mechanisms underlying sepsis-induced cardiomyopathy (SIC) remain poorly understood, and there are no specific therapeutics for SIC. We investigated the effects of maresin conjugates in tissue regeneration 1 (MCTR1) on SIC and explored its potential mechanisms. The experiments were conducted using an endotoxemia model induced by lipopolysaccharide (LPS). Mice were given MCTR1 intravenously 6 h after LPS stimulation. Echocardiography was performed to assess cardiac function 12 h after LPS administration. Treatment with MCTR1 significantly enhanced cardiac function and reduced LPS-induced increase of mRNA expression levels of inflammation cytokines. Transcriptomic analysis indicated that MCTR1 inhibited neutrophil chemotaxis *via* the IL-17 signaling pathway. We confirmed that MCTR1 reduced the expressions of neutrophil chemoattractants and neutrophil infiltration in the LPS-stimulated hearts. MCTR1 also resulted in a considerable reduction in IL-17A production mainly derived from *γδ* T cells. Moreover, our results provided the first evidence that neutralizing IL-17A or depletion of *γδ* T cells markedly decreased neutrophil recruitment and enhanced cardiac function in LPS-induced cardiac injury. These results suggest that MCTR1 alleviates neutrophil infiltration thereby improves cardiac function in LPS-induced cardiac injury *via* the IL-17 signaling pathway. Thus, MCTR1 represented a novel therapeutic strategy for patients with SIC.

## Introduction

Sepsis is defined as a lethal organ dysfunction caused by a dysregulated host response to infection (1). The heart is one of the most vulnerable organs in sepsis. Sepsis-induced cardiac dysfunction, also called sepsis-induced cardiomyopathy (SIC), has been summarized as a global (systolic and diastolic) but reversible dysfunction of both the left and right sides of the heart (2–4). Despite remarkable scientific and clinical efforts, the mechanisms underlying the myocardial dysfunction in sepsis are still not fully understood, and there are no specific therapeutics for SIC (3).

The pathophysiologic cascade of sepsis begins when the host immune system responds to an invading pathogen, resulting in the immune response activation. Interleukin-17A (IL-17A) is one of the members of the IL-17 family. Compared with other members, IL-17A plays a more prominent role in the mammalian immune system (5–7). IL-17A is a critical mediator of neutrophil recruitment and migration through the induction of granulopoiesis and the production of neutrophil chemokines, including granulocyte colony-stimulating factor (G-CSF), chemokine (C-X-C motif) ligand 1 protein (CXCL1), and chemokine (C-X-C motif) ligand 2 protein (CXCL2) (8). Although IL-17A exerts a host-defensive role in many infectious diseases, it promotes inflammatory pathology in auto-immunity and other settings (9). Dysregulated IL-17A production or uncontrolled response to IL-17A signaling promotes pathogenic inflammation (10). In the mouse model of myocardial ischemia–reperfusion injury, IL-17A was mainly produced by *γδ* T cells, and blockade of IL-17A significantly reduced neutrophil infiltration in the heart and alleviated cardiac injury (11, 12). Moreover, anti-IL-17A could protect the lungs in lipopolysaccharide (LPS)-induced acute lung injury and improve survival in polymicrobial sepsis induced by cecal ligation and puncture (CLP) (13–15). However, the role of IL-17A in sepsis-induced cardiac dysfunction is not clear.

Specialized pro-resolving mediators (SPMs) are enzymatically derived from essential fatty acids and have crucial roles in restoring tissue homeostasis during tissue inflammation (16). SPMs are distinct from immunosuppressive molecules as they not only dampen inflammation but also promote host defense (17). SPMs are partly defined by their overlapping functions as limiting neutrophil tissue accumulation, counter-regulating pro-inflammatory cytokines, and encouraging macrophage phagocytosis (18). Previous investigations indicated that the IL-17 signaling pathway might involve the inflammation resolving work of SPMs in myocardial infarction and allergic airway inflammation (19–21). In our previous study, we found that maresin conjugates in tissue regeneration 1 (MCTR1), a newly identified SPM, could reduce lipopolysaccharide (LPS)-induced cardiac injury by upregulating mitochondrial biosynthesis and improve the survival rate (22). The mechanism of SPMs on sepsis-induced cardiac dysfunction is not clear, and whether the IL-17 signaling pathway is engaged is also unknown. Therefore, in this study, we tried to clarify the mechanism by which MCTR1 reversed sepsis-induced cardiac dysfunction. We also verified the role of IL-17A in sepsis-induced cardiac dysfunction and confirmed whether IL-17A participated in the effect of MCTR1 on sepsis-induced cardiac dysfunction.

## Materials and Methods

### Animals

All animal care and experimental protocols complied with the Guide for the Care and Use of Laboratory Animals of the National Institutes of Health (NIH Publication 8th edition, 2011). Eight-to-twelve-week-old male C57BL/6 mice (Shanghai Experimental Animal Center of China) were used in this study, and the weight of mice was around 25 g. All these mice were housed at four per cage and maintained in a specific pathogen-free room with controlled temperature (23 ± 1°C) and humidity (55 ± 5%) under a 12 h light/dark cycle. The mice were given standard laboratory chow and water *ad libitum*. All animal experiments were approved by the Animal Studies Ethics Committees of the Second Affiliated Hospital of Wenzhou Medical University.

### Experimental Procedures

To evaluate the effects of MCTR1 on cardiac after LPS stimulation, the mice were randomly divided into four groups: saline control, LPS, LPS plus MCTR1, and MCTR1 along groups. The mouse model of endotoxemia was induced by an intraperitoneal injection of 0.2 ml of sterile saline containing LPS (10 mg/kg, serotype 055: B5; Sigma, Saint Louis, MO, USA). The control mice received an injection of saline in the same volume and route. MCTR1 was obtained from Cayman Chemical (Ann Arbor, MI, USA). MCTR1 was dissolved in ethanol supplied by the manufacturer and was stored at −80°C. Ethanol was blown away by nitrogen before use, and MCTR1 was dissolved rapidly in sterile saline to the desired concentration. In the MCTR1 groups, mice received MCTR1 (0.15 nmol/mouse) intravenously *via* the caudal vein 6 h after LPS stimulation as previously described (22). The dose of MCTR1 was selected based on previous studies (22, 23).

To evaluate the role of the *γδ* T cells and IL-17A in the cardiac dysfunction after LPS stimulation, neutralization of endogenous IL-17A and depletion of *γδ* T cells were performed. Neutralization of endogenous IL-17A as previously described (12, 24), 100 μg anti-mouse IL-17A antibody (CAT: MAB421, R&D System, Minneapolis, MN, USA) or 100 μg isotype control antibody was administered intravenously 5 min before LPS treatment. Mice were depleted of *γδ* T cells as previously described (14, 25). Five days before treatment with LPS, 500 μg Ultra-LEAFTM Purified anti-mouse TCR*γδ* antibody (CAT: 107517, BioLegend, San Diego, CA, USA) was administrated to mice by intraperitoneal injection. Sham depletion mice received equal amounts of isotype control antibodies. For tissue collection, mice were anesthetized by overdose pentobarbital sodium (100 mg/kg intraperitoneal injection) and then sacrificed by bloodletting from the abdominal aorta at 12 h after LPS treatment.

### Echocardiography

Echocardiography was performed with a Vevo 3100 instrument (Visual Sonics, Toronto, ON, Canada) as described previously (12, 26). Mice were anesthetized with 1.2% isoflurane. Left ventricular end-diastolic volume (LVEDV) and left ventricular end-systolic volume (LVESV) were evaluated using B-mode configuration. Left ventricular ejection fraction (EF) was calculated using the following formula: EF = [(LVEDV − LVESV)/LVEDV] × 100%. Left ventricular end-diastolic diameter (LVEDD) and Left ventricular end-systolic diameter (LVESD) were measured from M-mode tracing. Left ventricular fractional shortening (FS) was calculated using the following formula: FS = [(LVEDD − LVESD)/LVEDD] × 100%.

### RNA-Seq

RNA-Seq was performed by Aksomics (Shanghai, China). Significance of differentially expressed genes from transcriptome data was statistically determined with moderated t-test (*p*-value < 0.05), and false discovery rate (FDR; < 0.05%). The statistically significant genes were submitted to DAVID version 6.8 software (http://david.abcc.ncifcrf.gov) for gene ontology (GO) analysis. Functional pathways were selected in the KEGG database (Kyoto encyclopedia of genes and genomes, https://www.kegg.jp). Significantly enriched GO or functional pathways for up-regulated (UP) and down-regulated (DOWN) groups were visualized with –log10 transformation of *p*-value.

### Western Blotting Analysis

Tissues from the left ventricular were lysed in a RIPA lysis buffer with PMSF. The supernatants were quantified using the bicinchoninic acid (BCA) method. Then 30 μg denatured protein samples were separated by 10–12% SDS-polyacrylamide gel and transferred onto PVDF membranes (Millipore, Billerica, MA, USA). After blocking with 5% skimmed milk in TBST at room temperature for 2 h, the membranes were probed overnight at 4°C with one of the following primary antibodies: CXCL1 (1:1,000, Affinity Biosciences, Changzhou, China) and G-CSF (1:1,000, Bioss, Beijing, China). The membranes were then washed off of excess antibody and incubated with horseradish peroxidase-linked secondary antibodies (1:3,000) at room temperature for 1 h. After washing with TBST, the specific bands were visualized using the chemiluminescence detection system and analyzed with the AlphaEaseFC software (Alpha Innotech, San Leandro, CA, USA).

### ELISA

IL-17A in mouse serum was determined using a mouse IL-17A enzyme-linked immunosorbent assay (ELISA) kit (Boyun biotech, Shanghai, China) according to the manufacturer’s instructions.

### Immunofluorescence

Immunofluorescence analysis was performed on the paraffin-embedded sections. After deparaffinization, rehydration, and antigen retrieval with sodium citrate (pH 6.0), the tissue sections were blocked with donkey serum at room temperature for 1 h. Subsequently, the tissue sections were incubated with an anti-CXCL1 primary antibody (1:100, Affinity Biosciences, Changzhou, China) or an anti-Ly6G primary antibody (1:100, R&D Systems, Minneapolis, MN, USA) overnight at 4°C. Secondary antibodies coupled to Alexa Fluor 594 fluorophores (1:200) were then used and applied for 1 h at room temperature. Nuclei were stained with DAPI. Finally, tissue sections were observed with a Zeiss fluorescence microscope (Carl Zeiss AG, Oberkochen, Germany). A specific region of interest (ROI) was analyzed using ImageJ (version 1.38×, NIH, Bethesda, MD, USA) based on previous report (27).

### Quantitative PCR

Total RNA was extracted from mouse left ventricular tissues using TRIzol Reagent (Invitrogen, Carlsbad, CA, USA). Complementary DNA was synthesized from 1 μg RNA by reverse transcription kit (Thermo Scientific, Rockford, IL, USA). SYBR Green Real-time PCR Master Mix (Toyobo, Osaka, Japan) was used for real-time PCR. Gene expression levels were normalized with GAPDH as the housekeeping gene, and the expression changes were calculated using the 2^−△△^Ct method. All primer sequences were summarized in **Table 1**.

**Table 1:** Primer sequences for quantitative PCR.

Gene	Gene bank no.	Forward (5′–3′ sequence)	Reverse (5′–3′ sequence)
Nppb	NM_001287348.1	GAAGGACCAAGGCCTCACAA	ACTTCAGTGCGTTACAGCCC
Tnf	NM_001278601.1	CCCTCACACTCACAAACCAC	ACAAGGTACAACCCATCGGC
Il1b	NM_008361.4	TGCCACCTTTTGACAGTGATG	TGATGTGCTGCTGCGAGATT
Il6	NM_031168.2	TGATGTGCTGCTGCGAGATT	CGCACTAGGTTTGCCGAGTA
ccl2	NM_011333.3	TGCCCTAAGGTCTTCAGCAC	AAGGCATCACAGTCCGAGTC
ccl7	NM_013654.3	GGTCACGCCTAAGGAATGGT	GGGGGAGAATTCTGCAGCTAA
cxcl1	NM_008176.3	ACTCAAGAATGGTCGCGAGG	GTGCCATCAGAGCAGTCTGT
G-csf	NM_009971.1	CAGCCCAGATCACCCAGAATC	GCTGCAGGGCCATTAGCTTC
Il17a	NM_010552.3	GCTGACCCCTAAGAAACCCC	GAAGCAGTTTGGGACCCCTT
Gapdh	NM_001289726.1	GGGTCCCAGCTTAGGTTCAT	GGGACGAGGAAACACTCTCC

### Flow Cytometric Analysis

Single-cell suspensions were prepared as described previously (12, 28). Briefly, mice were deeply anesthetized and intracardially perfused with 20 ml of ice-cold PBS to eliminate blood cells. The hearts were minced with fine scissors and placed into a cocktail of 1 mg/ml collagenase II (Worthington, Lakewood, NJ, USA), 100 U/ml elastase (Worthington), and 100 U/ml DNase I (Sigma-Aldrich) and shaken at 37°C for 1 h. Tissue samples were then triturated through a 70 μm cell strainer and centrifuged (5 min, 400 g, 4°C). The obtained cells were counted after erythrocyte lysis and washed using PBS for further analysis. For staining, 5 × 10^6^ cells were pre-incubated for 5 min on ice with anti-CD16/CD32 antibody (2.4G2, BD Bioscience, San Jose, CA, USA) to block the non-specific antibody and then stained with directly conjugated antibodies for 30 min at 4°C in the dark in PBS. For intracellular cytokine staining, single-cell suspensions were stimulated with 50 ng/ml (PMA, Sigma-Aldrich), 1 μg/ml ionomycin (Sigma-Aldrich), and Golgi-PlugTM (BD Biosciences) for 4 h. Surface staining was performed first. After fixation and permeabilization using the Cytofix/Cytoperm Soln kit (BD Biosciences), intercellular proteins were stained. All experiments were performed on an Attune NxT flow cytometer (Invitrogen) and analyzed using FlowJo version 10 software.

### Myeloperoxidase Activity

The heart tissues were weighed and homogenized in the homogenate medium supplied by the myeloperoxidase (MPO) test kit (Jiancheng, Nanjing, China). We determined the MPO activity according to the manufacturer’s instructions.

### Statistics

Data are represented as mean ± standard deviation (SD). All data were analyzed by one-way analysis of variance followed by Tukey’s *post hoc* test for multiple comparisons. *P*-values <0.05 were considered statistically significant. Statistical analyses were performed using GraphPad Prism 7.0 software (GraphPad Software, San Diego, CA).

## Results

### Post-Treatment With MCTR1 Improved Cardiac Function in LPS-Induced Cardiac Injury

**Figure 1:**
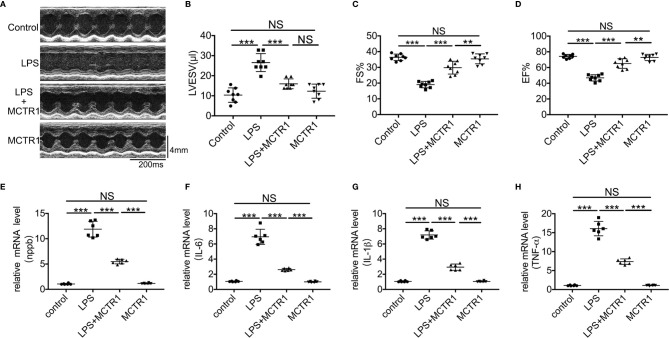
The effect of MCTR1 on cardiac function in LPS-challenged mice. **(A)** Representative M-mode echocardiographic recordings. **(B–D)** Left ventricular end-systolic volume (LVESV), fractional shortening (FS), and ejection fraction (EF) were analyzed (n = 8). **(E–H)** Nppb, Tnf-α, IL-1β, and IL-6 mRNA expressions were examined by quantitative PCR in the hearts (n = 6). Data are shown as means ± SD. **P < 0.01; ***P < 0.001; NS, not significant.

We previously demonstrated that cardiac function was decreased after LPS administration and peaked at 6 and 12 h after challenge (22). To verify whether MCTR1 can promote cardiac function recovery from damages of LPS, mice received MCTR1 6 h after administration with LPS. Then cardiac function was determined using echocardiography in another 6 h, that is 12 h after LPS administration (**Figure 1A**). The results in this study revealed that the left ventricular end-systolic volume (LVESV) significantly increased after the application of LPS. Post-treatment with MCTR1 markedly attenuated the increase of LVESV induced by LPS (**Figure 1B**). LPS significantly decreased left ventricular fractional shortening (FS) and ejection fraction (EF), which could be notably recuperated by MCTR1 without any effects on baseline cardiac function, and the values of FS and EF in the LPS + MCTR1 group were significantly lower than in the MCTR1 alone group (**Figures 1C, D**). These results indicated that MCTR1 could partly improve cardiac function in LPS-induced cardiac injury. In accordance with this, MCTR1 significantly reduced the mRNA expression level of natriuretic peptide B (nppb) which was up-regulated by LPS (**Figure 1E**). MCTR1 treatment markedly attenuated LPS-induced increase of mRNA expression levels of inflammation mediators as well, while the mRNA expressions of those mediators were significantly higher in the LPS + MCTR1 group than in the MCTR1 alone group (**Figures 1F–H**).

### RNA-Seq Analysis

**Figure 2:**
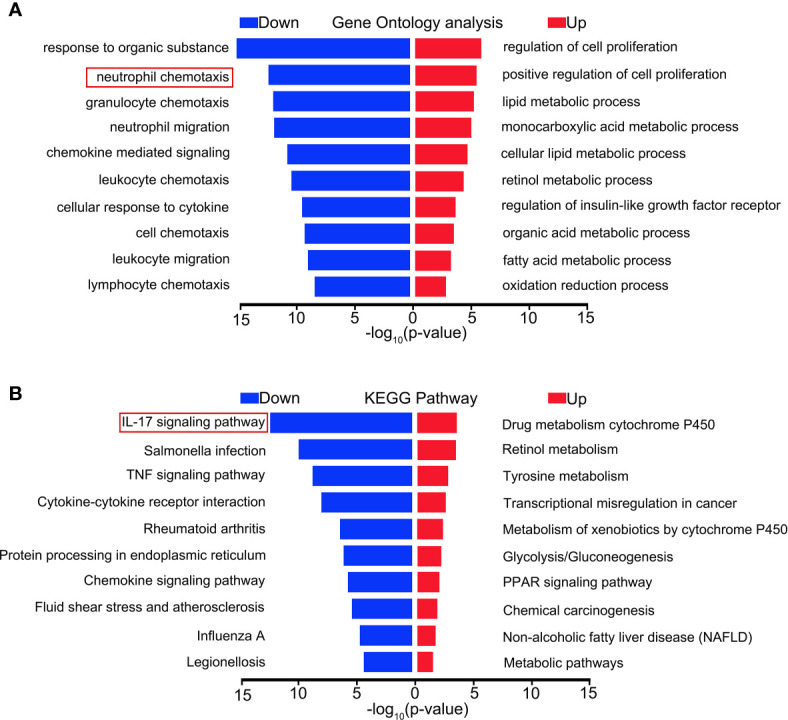
Distinct transcriptional signature between LPS and LPS + MCTR1 groups. 12 h after LPS treatment, the hearts were harvested and the transcriptome were analyzed by RNA Sequencing (RNA-Seq) technology between LPS and LPS + MCTR1 groups. Significance of differentially expressed genes was divided into UP- and DOWN-regulated gene lists. UP and DOWN genes were submitted for gene ontology (GO) analysis **(A)** and Kyoto encyclopedia of genes and genomes (KEGG) pathway enrichment analyses **(B)**.

To explore the mechanism of the effect of MCTR1 on the heart in LPS-induced endotoxemia, we performed RNA sequencing-based transcriptomic profiling of RNA isolated from cardiac tissues derived from LPS group and LPS + MCTR1 group in triplicate. Gene expression profiles of the two groups were evaluated by gene ontology (GO) and functional pathways (Kyoto encyclopedia of genes and genomes, KEGG) analysis. Genes were mostly enriched in the following top five GO groups (**Figure 2A**): response to organic substance, neutrophil chemotaxis, granulocyte chemotaxis, neutrophil migration and chemokine mediated signaling. KEGG pathway enrichment analysis demonstrated that the IL-17 signaling pathway was the most significantly affected pathway (**Figure 2B**).

### MCTR1 Repressed the Expressions of Neutrophil Chemokines

**Figure 3:**
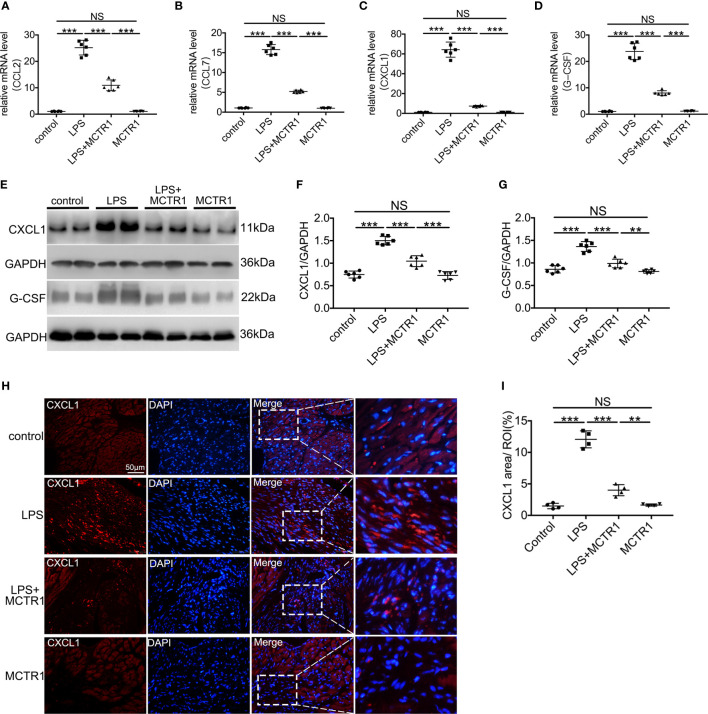
The effect of MCTR1 on chemokines expression in cardiac injury after LPS administration. **(A–D)** mRNA expression levels of CCL2, CCL7, CXCL1 and G-CSF were examined using quantitative PCR in the hearts (n = 6). **(E–G)** Protein expression levels of CXCL1 and G-CSF were detected by western blotting. Densitometry ratios of target proteins to loading control GAPDH were obtained (n = 6). **(H)** The Representative immunofluorescence staining of CXCL1 in the hearts. **(I)** Fluorescence intensity of CXCL1 was determined by using Image J software (n = 4). Data are shown as means ± SD. **P < 0.01; ***P < 0.001; NS, not significant.

We first verified several chemokines that changed significantly in transcriptomic results using quantitative PCR. The mRNA expression levels of CCL2, CCL7, CXCL1, and G-CSF exhibited remarkably enhancement in the LPS group compared with the control group. MCTR1 significantly attenuated the increase of these chemokines induced by LPS. Among them, the expression levels of CXCL1 and G-CSF, well-known neutrophil chemoattractants, changed most obviously (**Figures 3A–D**). Next, we used western blot to determine the protein expression levels of CXCL1 and G-CSF. The results revealed that MCTR1 dramatically down-regulated the CXCL1 and G-CSF in protein expression levels which were increased in the LPS group, while MCTR1 alone did not affect both of them (**Figures 3E–G**). Immunofluorescence analyses of cardiac tissues confirmed that CXCL1 mainly expressed in the LPS-injured hearts with very low expression in the non-LPS challenge hearts, and in line with aforementioned results, MCTR1 could considerably lessened the expression of CXCL1 induced by LPS, while the level of CXCL1 was significantly higher in the LPS + MCTR1 group than in the MCTR1 alone group (**Figures 3H, I**).

### MCTR1 Attenuated Neutrophil Infiltration in LPS-Stimulated Hearts

**Figure 4:**
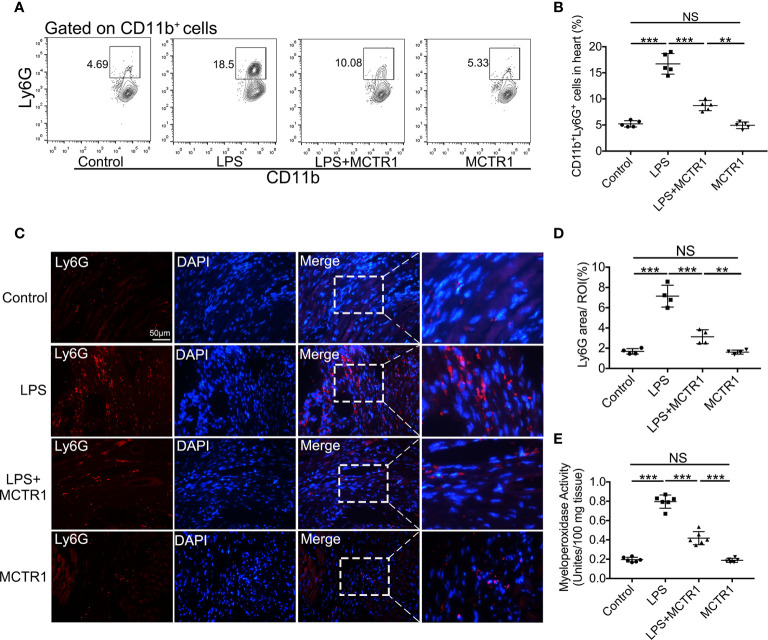
MCTR1 reduced neutrophils infiltration in LPS-induced cardiac injury. **(A)** The representative flow cytometry dot plots of CD11b^+^Ly6G^+^ neutrophils infiltrated in myocardium. **(B)** Percentage of CD11b^+^Ly6G^+^ neutrophil population in the hearts (n = 4). **(C)** Representative immunofluorescence staining of Ly6G in hearts. **(D)** Fluorescence intensity of Ly6G was determined by Image J software (n = 4). **(E)** Cardiac MPO activity was examined by MPO test kit (n = 6). Data are shown as means ± SD. **P < 0.01; ***P < 0.001; NS, not significant.

Consistent with increased chemoattractants, LPS induced a surge in neutrophil recruitment to the myocardium, and MCTR1 dramatically attenuated neutrophil recruitment induced by LPS as determined by flow cytometric analysis of CD11b^+^Ly6G^+^ neutrophils; neutrophil ratio was significantly higher in the LPS + MCTR1 group than in the MCTR1 alone group (**Figures 4A, B**). Immunofluorescence analyses of Ly6G expression and MPO activity determination confirmed that neutrophil infiltration was increased in the LPS group *versus* control group, and MCTR1 significantly reduced neutrophil infiltration in the LPS-stimulated hearts, Ly6G expression and MPO activity were significantly higher in the LPS + MCTR1 group than in the MCTR1 alone group (**Figures 4C–E**).

### MCTR1 Alleviated the Expression of IL-17A in LPS-Stimulated Hearts

**Figure 5:**
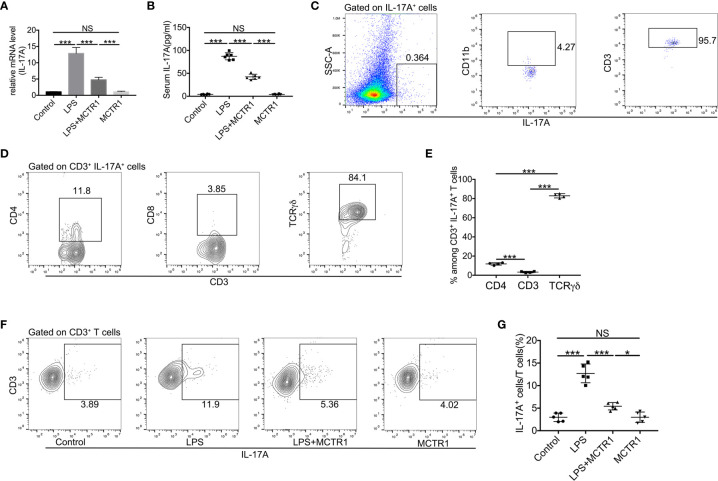
MCTR1 attenuated IL-17A production and IL-17A^+^ cells in LPS-induced cardiac injury. **(A)** The mRNA level of IL-17A in hearts was examined using quantitative PCR (n = 6). **(B)** IL-17A in serum was determined by ELISA (n = 6). **(C)** Flow cytometric analysis of CD11b and CD3 expressions on gated IL-17A^+^ cells in the LPS-damaged hearts. **(D, E)** TCR*γδ*, CD4, and CD8 expressions on gated CD3^+^IL-17A^+^ cells were examined by flow cytometric analysis in the LPS-damaged hearts (n = 4). **(F, G)** The percentage of IL-17A^+^ cells in all CD3^+^ T cells was determined by flow cytometric analysis (n = 5). Data are shown as means ± SD. *P < 0.05; ***P < 0.001; NS, not significant.

As the IL-17 signaling pathway may be involved in the effect of MCTR1 on cardiac function according to the transcriptomic analysis, and one of the prominent roles of IL-17A is neutrophil recruitment, we therefore first determined the expression of IL-17A by quantitative PCR and ELISA. The results revealed that LPS stimulation resulted in a significant increase in IL-17A production in the mouse hearts, which was markedly reduced in LPS + MCTR1 group, and the production of IL-17A was significantly higher in the LPS + MCTR1 group than in the MCTR1 alone group (**Figures 5A, B**). Accumulating evidence demonstrated that lymphocytes and innate myeloid immune cells are able to produce IL-17A (6). To clarify the cell source of IL-17A in the LPS-stimulation hearts, cardiac single cell suspension was stained and analyzed by flow cytometry. The results showed that *γδ* T cells were the dominant cells secreting IL-17A, but not CD4^+^ (Th17) or CD8^+^ T cells (**Figures 5C–E**). Treatment with MCTR1 resulted in a considerable reduction in IL-17A production from T cells in LPS challenged hearts, and IL-17A production was significantly higher in the LPS + MCTR1 group than in the MCTR1 alone group (**Figures 5F, G**). Collectively, these results indicated that MCTR1 alleviated neutrophil infiltration by reducing the production of IL-17A mainly derived from *γδ* T cells in LPS-induced cardiac injury.

### Neutralization of Endogenous IL-17A or Depletion of *γδ* T Cells Protects Against LPS-Induced Cardiac Injury

**Figure 6:**
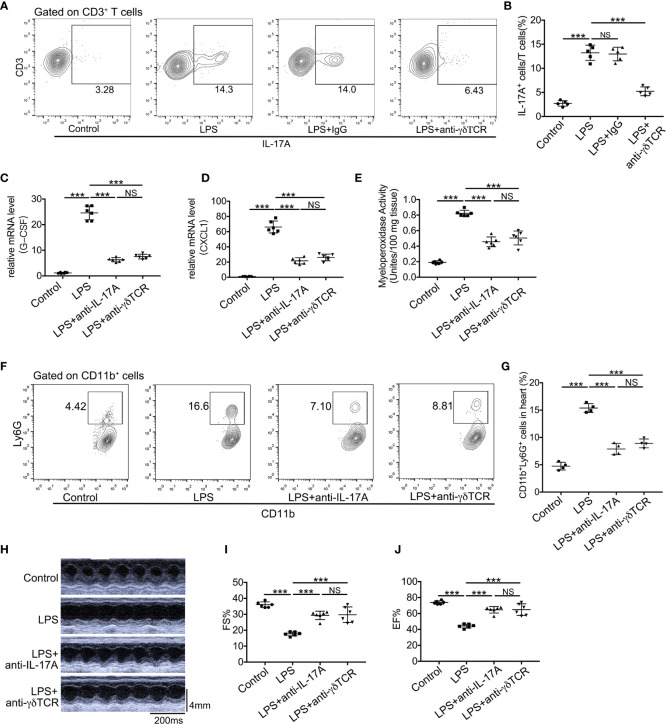
Neutralization of IL-17A or depletion of *γδ* T cells reduced neutrophil infiltration and improved cardiac function in LPS-induced cardiac injury. Cardiac function was determined using small animal ultrasound and cardiac tissues were collected 12 h after stimulation with LPS. **(A, B)** IL-17A^+^ cells in all CD3^+^ T cells were examined by flow cytometric analyses (n = 5). **(C, D)** mRNA levels of G-CSF and CXCL1 were analyzed using quantitative PCR (n = 6). **(E)** Cardiac myeloperoxidase activity was examined by MPO test kit (n = 6). **(F, G)** Flow cytometric analyses of the percentage of CD11b^+^Ly6G^+^ neutrophils in hearts (n = 4). **(H)** Representative cardiographic images of M-mode. **(I, J)**. Left ventricular FS and EF were determined by Echocardiographic analysis (n = 8). Data are shown as means ± SD. ***P < 0.001; NS, not significant.

The roles of IL-17A and *γδ* T cells in LPS-induced cardiac injury are still not elucidated, and we have demonstrated that IL-17A was mainly derived from *γδ* T cells in LPS-induced cardiac injury. To verify the effect of IL-17A derived from *γδ* T cells, we first depleted *γδ* T cells in mice by administrating anti-TCR*γδ* antibody and observed that the production of IL-17A in these *γδ* T cells depletion mice robustly decreased after LPS treatment (**Figures 6A, B**). We next analyzed mRNA expression levels of neutrophil chemoattractants upon neutralizing IL-17A or depleting *γδ* T cells in endotoxemia. As shown in **Figures 6C, D**, either neutralization of IL-17A or depletion of *γδ* T cells could significantly reduce mRNA levels of CXCL1 and G-CSF. Likewise, neutralizing IL-17A markedly decreased neutrophil infiltration as well as depletion of *γδ* T cells as determined by MPO activity and flow cytometry analysis in LPS-induced cardiac injury (**Figures 6E–G**). There was no significant difference in CXCL1 and G-CSF mRNA levels, MPO activity, and neutrophil ratio between the LPS + anti-IL-17A group and the LPS + anti-TCR*γδ* group (**Figures 6C–G**). To assess whether the neutralization of IL-17A or depletion of *γδ* T cells reversed the cardiac function inhibition caused by the general inflammation, we analyzed LVFS and LVEF 12 h after LPS administration. Neutralization of IL-17A could improve cardiac function to the same extent as depletion of *γδ* T cells (**Figures 6H–J**).

## Discussion

The present study investigated the effects and the mechanisms involved in the post-treatment with MCTR1 in the LPS-induced cardiac injury. Our results revealed that post-treatment with MCTR1 after intraperitoneal injection of LPS enhanced cardiac function and decreased the inflammation mediators in gene and protein levels. According to the transcriptome analysis, we confirmed that MCTR1 caused the reduction of neutrophil chemoattractants levels and the alleviation of neutrophil infiltration. MCTR1 also inhibited the production of IL-17A, which is mainly derived from *γδ* T cells in LPS-injured heart. In addition, our study added novel findings that neutralization of IL-17A or depletion of *γδ* T cells significantly ameliorated LPS-induced cardiac injury, which was associated with a reduction of neutrophil infiltration and improvement of cardiac function.

Patients with SIC typically exhibit ventricular dilatation, reduced ventricular contractility, and/or both right and left ventricular dysfunction with a reduced response to volume infusion (2, 29). We previously demonstrated that cardiac function in mice decreased most significantly at 6 and 12 h after intraperitoneal injection of LPS, and then gradually recovered (22). Here, we found that post-treatment with MCTR1 6 h after LPS treatment markedly reduced the left ventricular end-systolic volume and increased the left ventricular fractional shortening, left ventricular ejection fraction. Under this, MCTR1 inhibited LTD4-induced adverse inotropic action in isolated Ciona intestinalis (sea squirt) primordial hearts (23). In the LPS-induced acute lung injury, MCTR1 alleviated lung injury by protecting lung endothelial glycocalyx (30). In an animal model of myocardial infarction (MI), another SPM, RvD1 reduced neutrophil infiltration in ventricular and attenuated inflammation, thereby leading to the improvement of cardiac function (21).

Following cardiac injury, neutrophils lead the first wave of host defense to infection or tissue damage. Neutrophils are essential for the initiation of inflammation, resolving, and cardiac repair (31, 32). However, excess infiltration and activation of neutrophils lead to collateral damage in the myocardium (33). After the onset of inflammation, neutrophils and macrophages produce a series of SPMs, including lipoxins, resolvins, protectins, and maresins (16). These SPMs inhibit the excessive infiltration of neutrophils and help orchestrate the return to homeostasis (34–36). Transcriptome profiling in this study suggested that MCTR1 decreased neutrophil infiltration in which the IL-17 signaling pathway involved, and we confirmed that MCTR1 reduced the expression levels of G-CSF and CXCL1 which regulated neutrophil chemotaxis, decreased neutrophil recruitment in LPS-injured heart. IL-17 dominantly signals in non-hematopoietic cells (such as tissue-resident macrophages) to induce chemokines, including CXCL1, CXCL2, and CXCL8 (IL-8). These chemokines can attract neutrophils to infected or injured tissues. In addition, IL-17 induces G-CSF, which promotes the production and maturation of neutrophils from the bone marrow (33, 37). Our previous study revealed that MCTR1 promoted M2 polarization of resident macrophages *via* the STAT6 pathway to accelerate resolution of LPS-induced lung injury (38).

Previous studies revealed that the production of G-CSF and CXCL1 were regulated by IL-17A, which was primarily produced by *γδ* T cells and played a pathogenic role in myocardial I/R injury by inducing neutrophil infiltration (11, 12). These findings support our results that IL-17A was predominantly produced by *γδ* T cells but not CD4^+^ or CD8^+^ T cells in LPS-induced cardiac injury. IL-17A also contributed to the mechanisms of cardiac injury in the heart transplantation model (24, 39), angiotensin II-induced hypertensive heart injury (40), and myocarditis-induced cardiac fibrosis (41). The elevated plasma IL-17 level was associated with poor outcomes in post-cardiac arrest syndrome patients and left ventricular diastolic function in patients with diastolic heart failure (42, 43). Several clinical trials have been operated to evaluate the correlation between pro-inflammation mediators (IL-17) and sepsis (or SIC). However, the findings have been somewhat inconsistent. In critically ill children with severe sepsis, IL-17 showed a weak positive correlation with severity of illness and was significantly higher among non-survivors (44). Elevated serum IL-17 may increase the susceptibility for septic complications in polytrauma patients (45). However, in another preliminary study, the IL‐17/IFN pathway was associated with a faster sepsis resolution and a better survival (46). Here, we found that the level of IL-17A increased in the LPS-stimulated mice. In addition, our results provide the first evidence that neutralization of IL-17A or depletion of *γδ* T cells significantly down-regulated the G-CSF and CXCL1 levels, profoundly decreased neutrophil infiltration, and reversed the cardiac function in LPS-induce cardiac injury.

Our results also exhibited that post-treatment with MCTR1 reduced the IL-17A secretion in LPS-induced cardiac injury. Consistent with our results, previous studies have demonstrated that some SPMs could promote inflammation resolution by targeting at IL-17 signaling pathway. For example, resolvin E1 and resolvin E3 alleviated allergic airway inflammation by inhibiting the production of IL-17A (19, 20). Maresin 1 reduced IL-17A production by *γδ*TCRmid^+^ and CD4^+^ T cells in imiquimod-induced skin inflammation (47). Thus, our findings indicated that IL-17A might get involved in the alternative function of MCTR1 on LPS-induced cardiac injury.

**Figure 7:**
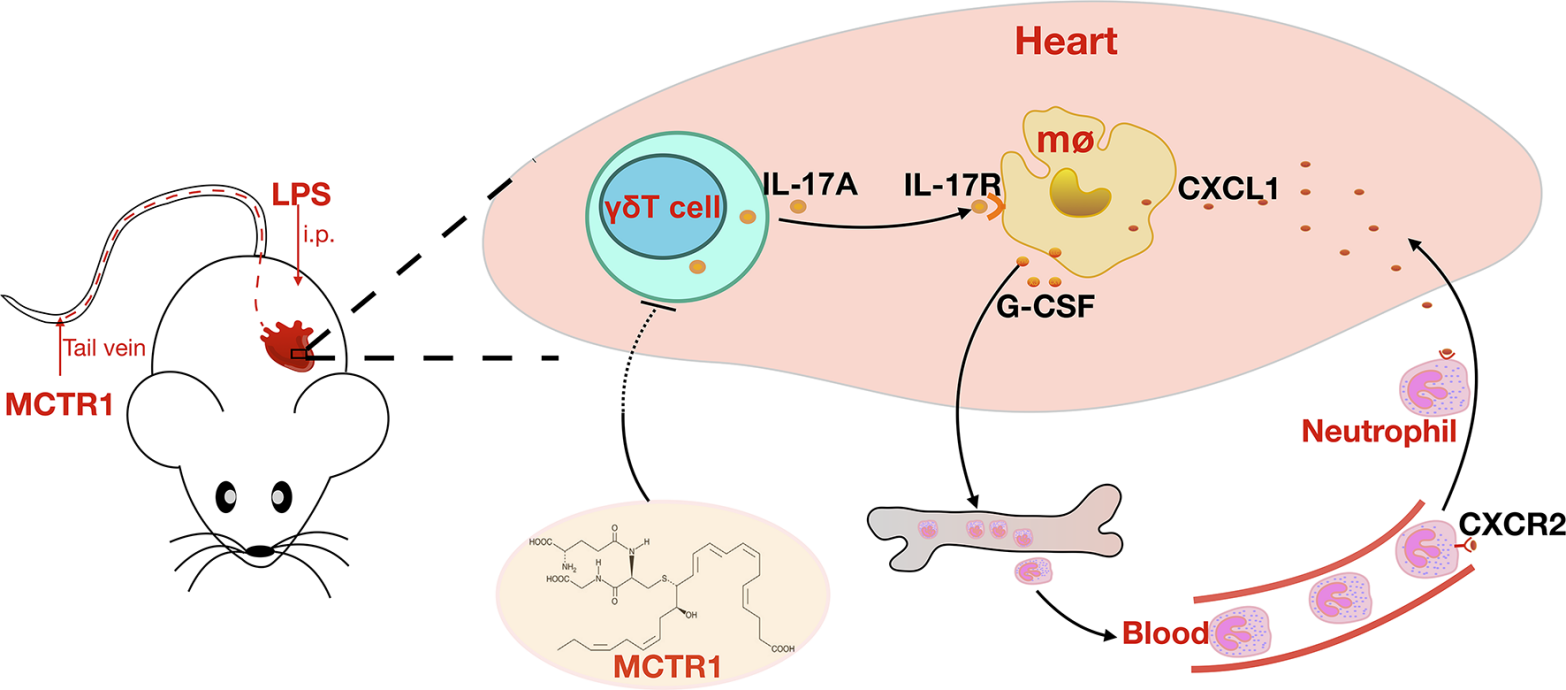
MCTR1 reduces neutrophil infiltration through attenuating IL-17A production derived from γδ T cells in LPS-induced cardiac injury.

In conclusion, our results provide insights into the protective role of MCTR1 in LPS-induced cardiac injury. MCTR1 alleviated the expression levels of neutrophil chemokines and neutrophil infiltration in the injured heart *via* the IL-17 signaling pathway (**Figures 7**). Thus, MCTR1 represented a novel therapeutic strategy for sepsis-induced cardiomyopathy.

## Data Availability Statement

The datasets presented in this study can be found in online repositories. The names of the repository/repositories and accession number(s) can be found in the article/supplementary material.

## Ethics Statement

The animal study was reviewed and approved by the Animal Studies Ethics Committees of the Second Affiliated Hospital of Wenzhou Medical University.

## Author Contributions

YY and J-GW conceived and designed the study. YY, X-YL, L-CL, Y-MZ, and YT performed the experiments. JX, YC, and Y-MS analyzed and validated the data. YY, S-WJ, and J-GW reviewed and edited the manuscript. All authors contributed to the article and approved the submitted version.

## Funding

This work was supported by the National Natural Science Foundation of China (81870065), Key Research and Development Project of Zhejiang Province (2019c03011) and Major Science and Technology Innovation Project of Wenzhou (2018ZY006).

## Conflict of Interest

The authors declare that the research was conducted in the absence of any commercial or financial relationships that could be construed as a potential conflict of interest.
